# Proprotein Convertase Subtilisin/Kexin Type 9 (PCSK9) Gene Is a Risk Factor of Large-Vessel Atherosclerosis Stroke

**DOI:** 10.1371/journal.pone.0001043

**Published:** 2007-10-17

**Authors:** Shérine Abboud, Pekka J. Karhunen, Dieter Lütjohann, Sirkka Goebeler, Teemu Luoto, Silvia Friedrichs, Terho Lehtimaki, Massimo Pandolfo, Reijo Laaksonen

**Affiliations:** 1 Laboratory of Experimental Neurology, Department of Neurology, Erasme Hospital, Universite Libre de Bruxelles, Brussels, Belgium; 2 School of Medicine, University of Tampere and Research Unit of the Laboratory Centre, Tampere University Hospital, Tampere, Finland; 3 Department of Clinical Pharmacology, University of Bonn, Bonn, Germany; 4 Laboratory of Atherosclerosis Genetics, Department of Clinical Chemistry, Tampere University Hospital and Medical School, University of Tampere, Finland; Amgen, United States of America

## Abstract

**Background/Purpose:**

Genetic variation in proprotein convertase subtilisin/kexin type 9 (PCSK9) gene has been recently identified as an important determinant of plasma LDL-cholesterol and severity of coronary heart disease. We studied whether the PCSK9 gene is linked to the risk of ischemic stroke (IS) and with the development of intracranial atherosclerosis.

**Methods/Results:**

The pivotal E670G polymorphism, tagging an important haplotype of the PCSK9 gene, was genotyped in two independent studies. The Belgium Stroke Study included 237 middle aged (45–60) Belgian patients, with small-vessel occlusion (SVO) and large-vessel atherosclerosis stroke (LVA), and 326 gender and ethnicity matched controls (>60 yrs) without a history of stroke. In multivariate analysis the minor allele (G) carriers appeared as a significant predictor of LVA (OR = 3.52, 95% CI 1.25–9.85; p = 0.017). In a Finnish crossectional population based consecutive autopsy series of 604 males and females (mean age 62.5 years), G-allele carriers tended to have more severe allele copy number-dependent (p = 0.095) atherosclerosis in the circle of Willis and in its branches.

**Conclusion:**

Our findings in this unique combination of clinical and autopsy data, provide evidence that PCSK9 gene associates with the risk of LVA stroke subtype, and suggest that the risk is mediated by the severity of intracranial atherosclerosis.

## Introduction

Familial predisposition has a modest effect (odds ratio (OR) 1.3–1.76) to the risk of stroke in general [1]. Due to heterogeneity of stroke, studies targeted on stroke subtypes could increase the possibility to reveal underlying genetic background of stroke. Earlier epidemiological studies have shown an increased genetic influence in small-vessel occlusion (SVO) and large-vessel atherosclerosis (LVA) stroke as defined by the Trial of org 10172 in acute treatment (TOAST) classification, particularly in relative young stroke patients (OR 2.5–4.5, <60 yrs) [2] [3] [4].

Proprotein convertase subtilisin/kexin type 9 (PCSK9), a serine protease, has recently gain a lot of attention because of its major role in regulation of plasma low density lipoprotein (LDL) cholesterol levels [5] [6] [7] [8] [9] [10] and in determining coronary heart disease (CHD) risk [6] [11] [12]. PCSK9 promotes degradation of the low density lipoprotein receptors (LDLR) in liver through an unknown posttranscriptional mechanism [13] In the large long-term Atherosclerosis Risk in Communities study, some sequence variations of the PCSK9 gene associated both with low LDL cholesterol levels and reduced incidence of coronary events [6]. On the other hand, some other sequence variants have associated with premature atherosclerosis development [12]. The Lipoprotein Coronary Atherosclerosis Study (LCAS) investigators identified the E670G variation as the most important tagging polymorphism of the PCSK9 gene that acted as an independent determinant of plasma LDL cholesterol levels and coronary atherosclerosis severity [9]. Furthermore, the G allele has been observed to relate to polygenic hypercholesterolemia in men [14].

In the present study we assessed the role of the E670G variation tagging an important haplotype of the PCSK9 gene as a possible risk factor for IS and its subtypes and we tested its association with the semi quantitative score of atherosclerosis of the circle of Willis and its branches in a large consecutive Finnish autopsy series.

## Materials and Methods

The Belgium Stroke Study (BSS) included 237 subjects with SVO and LVA stroke according to the TOAST classification occurring between 45 and 60 years of age. Among these patients 114 had SVO, 103 LVA, and 20 had SVO and LAA. They were selected from seven Stroke Units in Belgium. All patients were of central European origin (>90% were Belgians). Gender and ethnicity matched subjects (>60 years, n = 326) without a history of IS or CHD were recruited as controls from the general population, in order to avoid that the recruited controls would later turn out to be actually cases, we on purpose selected older controls. The optimal method of identifying and controlling for population stratification in genetic association studies is not known. A recent study showed that the grand parental country origin provided a better control for stratification than the SNP based approach. [15] In this study, the ethnicity was checked until the fourth grand parents. Cardiovascular risk factors (hypertension, diabetes mellitus, hyperlipidemia, alcohol consumption (>2 glasses of alcohol a day), smoking (former, current, never), obesity (body mass index (BMI) >30)) were recorded in cases and controls. The study protocol was approved by the ethical committees of all participating Belgium hospitals: Erasme Hospital, CHU Brugmann, and Cliniques Universitaires Saint-Luc in Brussels, CHC Clinique de l'Espérance of Montegné, Cliniques Universitaires of Mont-Godinne, CHU of Charleroi, and CHU of Tivoli. Informed written consent was obtained from all patients before study entry.

The Tampere Coronary Study (TCS) is a cross-sectional population based autopsy study comprising a total of 604 caucasian Finnish autopsy cases who had died suddenly out-of-hospital. The TCS included both men (64.3%, mean age 59.7) and women (35.7%, mean age 68.2). In each case, the atherosclerosis of each of the nine branches of the circle of Willis was scored semi-quantitatively (0 = normal, 1 = slight: streaks with or without elevated fibrous lesions, 2 = moderate: fibrous lesions that cause <50% stenosis, 3 = severe: >50 stenosis with extensive atherosclerosis (fatty, fibrous, calcified lesions)) giving a range of scores from 0 to 27). The study protocol was approved by the Board of Medicolegal Affairs of Finland. Informed written consent was obtained from relatives.

### Genotyping

In the BSS, DNA was isolated whole blood stored frozen at −20°C, with a commercial kit (Qiagen Inc. Valencia, CA)). In the TCS, DNA isolation was performed from frozen blood samples with the salt precipitation method. Genotyping was done by using the 5′ nuclease assay and fluorogenic allele-specific TaqMan MGB probes in the ABI Prism 7900 HT sequence detection. The nucleotide sequences of primers and probes used in the PCR of E670G (23,968A>G) (rs 505151) were deduced from public databases and synthesized in conjunction with Applied Biosystems.

### Statistical analysis

The data was analyzed with the SPSS software (version 12.0, SPSS Inc., Chicago, IL, USA).

The clinical data were compared between IS cases and controls, using chi-square tests for discrete variable. Logistic regression analysis with smoking, obesity, hypertension, alcohol consumption, diabetes, and hyperlipidemia as dichotomous variables was used to evaluate the association of E670G SNP with IS and its subtypes (SVO and LVA). The association between E670G and intracranial atherosclerosis was performed using a one-way ANOVA model, followed by an ANCOVA analysis by adding gender as dichotomous covariate and age, and BMI as continuous covariates in the model.

## Results

The clinical characteristic of cases and controls are presented in Table 1. As expected the patients had a higher prevalence of conventional cardiovascular risk factors than the controls (Table 1).

**Table 1:** Clinical characteristics of stroke patients and controls.

	Controls n = 326	IS n = 237	p-value
Mean age (y)	70.3	53.5	
Hypertension %	38.0	63.8	<0.001
Diabetes %	10.8	16.3	0.042
Hyperlipemia %	36.5	57.9	<0.001
Smoking status %			
Current	9.3	55.9	<0.001
Former	21.0	15.0	
Never	69.8	29.1	
Alcohol consumption (>2 glass/day) %	21.2	30.3	0.011
Familial history of MI or Stroke %	46.6	71.8	<0.001
Obesity (BMI>30) %	13.2	16.5	0.172
Sex (male) %	66.0	66.8	0.856

χ2 test

MI = myocardial infarction, BMI = body mass index

IS ischemic stroke

The genotype frequency distributions were in Hardy-Weinberg's equilibrium among cases and controls. The frequency of EE, EG and GG variants in the Belgium population were 94.1% 5.7% and 0.2%. Due to the rare occurrence of GG homozygotes, G allele carriers (EG+GG) were combined and compared to EE homozygotes. In a multivariate analysis the G allele tended to be more common among IS cases than controls (8.1% vs. 4.3%; p = 0.095). In particular, the G allele was significantly more common among LVA patients than in control subjects (10.8% vs. 4.3%; p = 0.017 OR 3.52, 95%CI 1.25–9.85*)* (Table 2). With a frequency of ∼6% for the at risk allele at an alpha level of 0.05, our sample was evaluated to have 80% power to detect a RR of 2.5 for heterozygote (“genetic power calculator”: http://statgen.iop.kcl.ac.uk/gpc/cc2.html). The E670G variation was not related to the risk of SVO (Table 2). As SVO and LVA were ad hoc determined scientifically reasonable variables correction for multiple testing was not primarily applied. However, Bonferoni corrected p-values are also given in table 2 for LVA.

**Table 2:** PCSK9 Genotype frequencies (%) for cases and controls.

PCSK9 genotype	Controls N = 326	All IS N = 237	p1	p2	OR (95%CI)	LVA N = 103	p1	p2	p3	OR (95%CI)	SVO N = 114	p1	p2	OR (95%CI)
EE	95.7	91.9	**0.047**	0.095	2.10 (0.87-5.05)	89.2	**0.019**	**0.017**	**0.034**	3.52 (1.25-9.85)	93.8	0.286	0.699	1.23 (0.37-4.11)
EG + GG	4.3	8.1				10.8					6.2			

Statistics: p1  = χ 2 test, p2 = Logistic regression analysis, with adjustment for: hypertension, hyperlipemia, diabetes, obesity, smoking, alcohol consumption

LVA = large-vessel atherosclerosis, SVO = small-vessel occlusion, IS = ischemic stroke, p3 = after controlling for multiple testing with Bonferoni correction.

**Figure 1:**
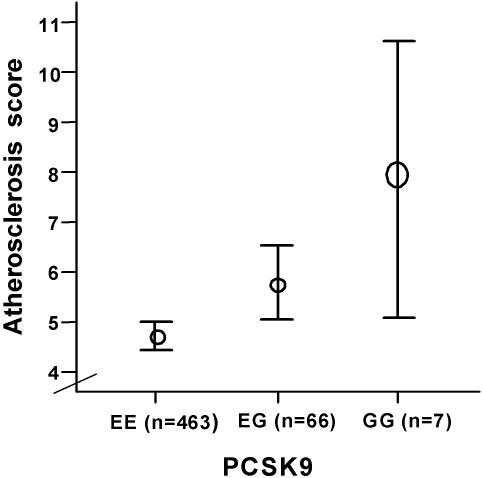
Effect of PCSK9 variants on the mean atherosclerosis score of the circle of Willis cerebral arteries. In the autopsy study, each of the nine branches of the circle of Willis was scored semi-quantitatively (0 = normal, 1 = slight: streaks with or without elevated fibrous lesions, 2 = moderate: fibrous lesions that cause<50% stenosis, 3 = severe:>50 stenosis with extensive atherosclerosis (fatty, fibrous, calcified lesions)) giving a range of scores from 0 to 27. Results are mean±SEM.

The frequency of EE, EG and GG variants in the Finnish autopsy series was 86.4%, 12.4% and 1.3%. Compared to carriers of the major EE genotype, G-allele carriers had more severe atherosclerosis in the large intracranial cerebral arteries (EE = 4.71 (CI 4.17–5.26)<G+ = 5.97 (CI 4.55–7.40) p = 0.095). There was an allele copy number-dependent trend for the mean atherosclerosis scores (EE = 4.71 (CI 4.17–5.26)<EG = 5.77 (CI 4.50–7.25)<GG = 7.86 (CI 1.12–14.60); p = 0.169). (Figure 1)

## Discussion

The main observation of this study was that the E670G SNP of PCSK9 gene associated significantly with LVA stroke risk in the Belgian population. The same allele tended to associate with increased atherosclerosis of the large intracerebral arteries in an independent Finnish autopsy study, with a gene dose effect.

The PCSK9 gene encodes proprotein convertase subtilisin-kexin type 9, a secreted enzyme of the serine protease family which plays a role in regulating LDL receptor concentration in liver. Its activation leads to decreased amounts of hepatic LDL receptors and consequently to higher levels of plasma LDL cholesterol [16] [17] [18]. Mutations in this gene have been associated with both hypocholesterolemia and hypercholesterolemia through “loss-of-function” [8] [19] [7] and “gain-of-function” [5] [20] [21] [12], respectively. In addition, this gene has been suggested to be involved in the risk for coronary heart disease [6] [11] [12], and to contribute to the severity of coronary artery atherosclerosis in the Lipoprotein Coronary Atherosclerosis Study **(**LCAS) study population [9].

In this study, we have demonstrated a potential role of this gene in cerebrovascular disease, especially in LVA stroke; which is related to atherothrombotic lesions within large intracranial arteries forming the circle of Willis in the brain base. This observation is further supported by the fact that the same variant tended to have a gene dose effect on atherosclerosis of the circle of Willis arteries in the autopsy cases. However, this association was not present with SVO which is caused predominantly by a diffuse disease in smaller arterioles (lypohyalinose) and to a lesser extent by atherosclerosis [22], Thus, PCSK9 seems to affect atherosclerosis development similarly in different vascular beds of similar calibre and determine Cerebrovascular LVA as well as CHD events.

The mechanism by which PCSK9 affects this phenotype could be related to its known effect on plasma LDL-cholesterol concentrations. It is also possible that PCSK9 itself may have a direct pro-atherogenic effect. This was suggested by Cohen et al (7), as they observed that protection against coronary heart disease risk was greater than expected by the effect of PCSK9 sequence variants on LDL cholesterol [6].

In conclusion, we found that the pivotal E670G polymorphism, tagging an important haplotype of the PCSK9 gene associates specifically with the risk of LVA stroke subtype, and tended to have a gene dose effect on the severity of atherosclerosis of the large intracranial arteries forming the circle of Willis. Based on earlier observations on the major effect of sequence variation of PCSK9 gene on plasma LDL cholesterol concentrations and coronary heart disease risk and the result of the current study, PCSK9 seems to be an interesting new target molecule for the development of new antiatherogenic therapies.
